# Investigation of the 2312 flexoelectric coefficient component of polyvinylidene fluoride: Deduction, simulation, and mensuration

**DOI:** 10.1038/s41598-017-03403-7

**Published:** 2017-06-09

**Authors:** Shuwen Zhang, Kaiyuan Liu, Minglong Xu, Hao Shen, Kai Chen, Bo Feng, Shengping Shen

**Affiliations:** 1 State Key Laboratory for Strength and Vibration of Mechanical Structures, School of Aerospace, Xi’an Jiaotong University, Xi’an, 710049 China; 2 Center for Advancing Materials Performance from the Nanoscale (CAMP-Nano), State Key Laboratory for Mechanical Behavior of Materials, Xi’an Jiaotong University, Xi’an, 710049 China

## Abstract

Flexoelectric effects hold promising applications in sensing, actuating, and energy capturing, and thus it is demanded to measure the flexoelectric coefficient tensors of dielectric materials accurately. In this work, an approach to measuring the effective flexoelectric coefficient tensor component *μ*_2312_ of polymeric materials is developed by imposing a torque load upon a half cylindrical specimen. It is proven that *μ*_2312_ can be calculated by assessing the electric charge on the axial plane and the strain gradient along the radial direction, both induced by the torque. To overcome the difficulty in experimental measurements, the relationship between the strain gradient and torque is deduced theoretically and further verified with finite element analysis. This approach is applied to testing bars machined from bulk polyvinylidene fluoride (PVDF). Potential errors from the piezoelectric effects and the non-uniform strain gradient are discussed to verify the validity of the measurement. The experimental results show good reproducibility and agreement with other measured effective flexoelectric tensor components of PVDF. This work indicates a potential application of PVDF-based mechanical sensors and provides a method to investigate the effective flexoelectric coefficient component of polymers.

## Introduction

Flexoelectricity describes the electric polarization induced by strain gradient in dielectric materials. The fourth rank tensor flexoelectric coefficient, *μ*_ijkl_, measures the electric polarization vector produced by a gradient of the 2^nd^ rank strain tensor, which is expressed by the following equation, considering the coupling of flexoelectricity and piezoelectricity:

1 $${P}_{l}={\mu }_{ijkl}\frac{\partial {\varepsilon }_{ij}}{\partial {x}_{k}}+{P}_{piezo},$$

where *P*_*l*_, *ε*_ij_, *x*_k_, *d*_*33*_ and *P*_*piezo*_ are the electric polarization, applied strain tensor, position coordinate, the piezoelectric induced polarization, respectively. The theoretical predictions and discussions on flexoelectricity in crystals have been given by Tagantsev *et al*. since 1980s^1–4^. According to the simplified model^5^, the flexoelectric coefficient is estimated to have a magnitude of *e*/*a* ≈ 10^−10^ C/m, where *e* is the elementary electric charge and *a* is the crystal lattice parameter. First principles theory has been employed to calculate the full flexoelectric coefficient tensors^6^. The effective flexoelectric coefficient tensor components of SiTrO_3_ are further discussed by Zubko *et al*.^7, 8^ Ma and Cross developed the cantilever beam bending and four-point bending approach, which were soon adopted and optimized by other researchers, to study the flexoelectric effects in crystalline materials such as BaSrTiO_3_^9^, BaTiO_3_^10^, BaSn_x_Ti_1−x_O_3_^11^, Pb(Mg_1/3_Nb_2/3_)O_3_^12^, and PbZr_1−x_Ti_x_O_3_^13^. The flexoelectric coefficients of these perovskite ceramics are measured to be four or five orders of magnitude higher than the simplified model, and therefore flexoelectricity based sensing devices are proposed and demonstrated^14–21^. However, such applications are greatly limited by the brittleness of ceramic materials. Polymers, on the other hand, have been proven to be an appealing alternative, for its combined excellent flexoelectric and mechanical properties^22–25^. Although its flexoelectric coefficient may be lower than the perovskite ceramics, it sustains much higher shear and bending strains than most of the bulk ceramics.

In order to deepen the understanding and advance the applications of the flexoelectricity in polymers, accurate and systematic measurements of flexoelectric coefficients are in great demand. It has been recognized that the flexoelectricity in polymers may be different and more complicated than that in inorganic crystals. Quang *et al*. deduced and specified the number and types of all possible rotational symmetries for the flexoelectric tensors in inorganic crystals^26^. Polymers, however, are composed of carbon chains, which are formed by strong carbon-carbon covalent bonds. Hydrogen, oxygen, nitrogen, fluorine, sulfur, and/or phosphorus atoms are linked with the carbons by polarized covalent bonds as well. Between different molecular chains, van der Waals forces exist, which are essential for the mechanical and physical properties of polymers but much weaker in strength than the intramolecular covalent bonds^27^. When imposed under the external deformation, intermolecular rotation, rather than classic displacive deformation, will take place easily under a relatively lower stress level, without changing the angles and lengths of the covalent bonds. Therefore, the centric symmetry breaking may not exist in polymers. Chu *et al*. studied the flexoelectricity of several thermoplastic and thermosetting polymers by testing their dielectric polarization responses under bending deformation^23^. Baskaran *et al*. proposed a giant flexoelectric coefficient measurement of polyvinylidene fluoride (PVDF) by exerting tensile load on a specimen with inhomogeneous section shape^24, 25^. In our previous work, experimental approaches have been developed to assessing the flexoelectric coefficient components *μ*_1211_ and *μ*_3121_ of polymers. As an example, these methods were applied to PVDF, and *μ*_1211_ and *μ*_3121_ were measured in the magnitude of 10^−10^ C/m and 10^−8^ C/m, respectively^28–30^. It is worth emphasizing that all those measured values are effective flexoelectric coefficients, for the following reasons. First of all, for most of the polymers, single crystals are hard to obtain. Therefore the subscripts of the coefficients, unlike the ones in inorganic crystals, do not represent specific crystalline directions. Instead, the subscripts are just reflecting the geometric parameters ^23–25^. Secondly, unlike inorganic crystals in which elastic and plastic strains can be clearly defined, the deformation mechanism in polymers, as stated previously, is more complicated, and thus the measured strain *ε*_ij_ is not directly related to the chemical bonding configurations.

In this work, we adopted the previous subscripts denotation method and propose a novel way to measure the effective shear flexoelectric coefficient tensor component *μ*_2312_ by imposing a torque upon a half cylindrical shaped specimen, and by setting the electrodes on the axial plane. By this means, the *μ*_2312_ component of PVDF was successfully measured. We believe this method is also applicable to other polymeric materials, and thus shed light on the design and fabrication of polymeric dielectric materials based mechanical sensing devices.

## Results

**Figure 1:**
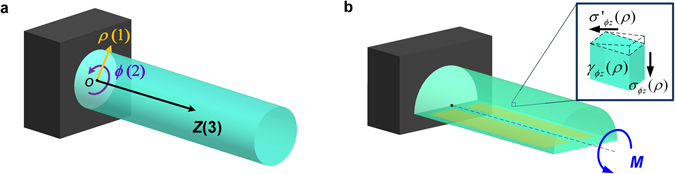
(**a**) The coordinate definition presented in this work. (**b**) The strain gradient generating method of the half cylindrical shaped specimen with the electrode setting.

To prove that the coefficient component we measured is *μ*_2312_, a cylindrical coordinate system is established, as shown Fig. 1a. The radial, azimuthal, and longitudinal directions are denoted as *ρ*, *ϕ*, and *z*, respectively. Based on Eqn. 1, to obtain the flexoelectric coefficient tensor component *μ*_2312_, a non-uniform shear strain *γ*ϕ_z_ needs to be generated, and its gradient along *ρ*direction should be measured. In the meanwhile, the electric polarization *P*ϕ along *ϕ*direction has to be monitored.

According to this analysis, a twisting moment *M* is imposed upon a continuous half-cylindrical solid, as indicated in Fig. 1b. The three-dimensional torsion can be simplified into plane stress analysis due to the constant section, according to the Saint Venant’s Principle^31, 32^. According to the Prandtl stress function, the shear stress meets:

2 $${\nabla }^{2}\psi =-2G\alpha ,$$

where *ψ* is the Prandtl stress function, *G* is the shear modulus, and *α* is the torque angle per unit length, which is assumed to be constant along the longitudinal axis.

Under the cylindrical coordinate system, Eqn. 2 is re-written as:

3 $$\frac{{\partial }^{2}\psi }{\partial {\rho }^{2}}+\frac{1}{\rho }\frac{\partial \psi }{\partial \rho }+\frac{1}{{\rho }^{2}}\frac{{\partial }^{2}\psi }{\partial {\varphi }^{2}}=-2G\alpha ,$$

with the boundary conditions of

4 $$\{\begin{array}{c}\psi (R,\varphi )=0\\ \psi (\rho ,0)=0\\ \psi (\rho ,\pi )=0,\end{array}$$

where *R* is the radius of the half cylinder.

Expanding the stress function and −2*Gα* to Fourier sine series, and applying the boundary conditions, the Prandtl stress function *ψ* is solved to be:

5 $$\psi (\rho ,\varphi )=\frac{8G\alpha }{\pi }\sum _{n=1,3,5,\mathrm{...}}^{\infty }\frac{{R}^{2}{(\frac{\rho }{R})}^{n}-{\rho }^{2}}{n(4-{n}^{2})}\,\sin (n\varphi ).$$

Therefore, the shear stress along the radial direction *τ*ϕ_z_ can be calculated from the stress function as

6 $${\tau }_{\varphi z}=\frac{1}{\rho }\frac{\partial \psi }{\partial \varphi }=\frac{8G\alpha }{\pi }\sum _{n=1,3,5,\mathrm{...}}^{\infty }\frac{{R}^{2}{(\frac{\rho }{R})}^{n}-{\rho }^{2}}{(4-{n}^{2})\rho }\,\cos (n\varphi ),$$

where *ϕ* equals to 0 or *π* for the plane under discussion, which is parallel with the longitudinal axis of the cylinder. Here we take the half plane that is under positive shear stress, therefore *ϕ* is 0 and cos(*nϕ*) = 1.

An advanced thermodynamic description is presented for the identification of the thermodynamically related converse effects and set up a base of a proper foundation for the research of energy stability of the specimen^33, 34^. In this description, the gradient of the electric polarization-induced stress contributes to the deformation of the material, and enhances the general deformation, especially in the dielectrics with high dielectric constants and in smaller scales. Considering the low dielectric constant, the macro-scale of the designed specimen, and the previously measured electric polarization magnitudes^28–30^, this coupling effect is negligible in this study, hence Hooke’s law is adopted for the analysis of the stress-strain relationship. In the elastic regime, the shear strain *γϕ*_z_ is proportional to the shear stress simply by applying the shear modulus *G* ^31, 32^

7 $${\gamma }_{\varphi z}=\frac{{\tau }_{\varphi z}}{G}.$$

Substituting Eqn. 6 into Eqn. 7, the shear strain gradient along the radial direction is calculated as

8 $$\frac{\partial {\gamma }_{\varphi z}(\rho )}{\partial \rho }=\frac{\partial [\frac{8\alpha }{\pi }\sum _{n=1,3,5,\mathrm{...}}^{\infty }\frac{{R}^{2}{(\frac{\rho }{R})}^{n}-{\rho }^{2}}{(4-{n}^{2})\rho }]}{\partial \rho }.$$

The torque angle *α* is related with the torsional moment *M* according to the following equation:

9 $$M=2\iint \psi \rho d\rho d\varphi =8G\alpha {R}^{4}\pi \sum _{n=1,3,5,\mathrm{...}}^{\infty }\frac{1}{{n}^{2}{(n+2)}^{2}{\pi }^{2}}.$$

We notice that $\sum _{n=1,3,5,\mathrm{...}}^{\infty }\frac{1}{{n}^{2}{(n+2)}^{2}}=\frac{{\pi }^{2}-8}{16}$, so that Eqn. 9 can be re-written as:

10 $$\alpha =\frac{2\pi M}{G{R}^{4}({\pi }^{2}-8)}.$$

Substituting Eqn. 10 into Eqn. 8 and replacing shear modulus *G* by Young’s modulus *E* and Poisson’s ratio *v*, it is deduced that the strain gradient can be calculated as:

11 $$\frac{\partial {\gamma }_{\varphi z}(\rho )}{\partial \rho }=\frac{32M(1+\nu )}{E{R}^{4}({\pi }^{2}-8)}\sum _{n=1,3,5,\mathrm{...}}^{\infty }\frac{(n-1){(\frac{\rho }{R})}^{n-2}-1}{4-{n}^{2}}.$$

For simplicity, Eqn. 11 is re-written as

12 $$\frac{\partial {\gamma }_{\varphi z}(\rho )}{\partial \rho }=c\cdot M,$$

where $$c=\frac{32(1+\nu )}{E{R}^{4}({\pi }^{2}-8)}\sum _{n=1,3,5,\mathrm{...}}^{\infty }\frac{(n-1){(\frac{\rho }{R})}^{n-2}-1}{4-{n}^{2}}.$$

**Figure 2:**
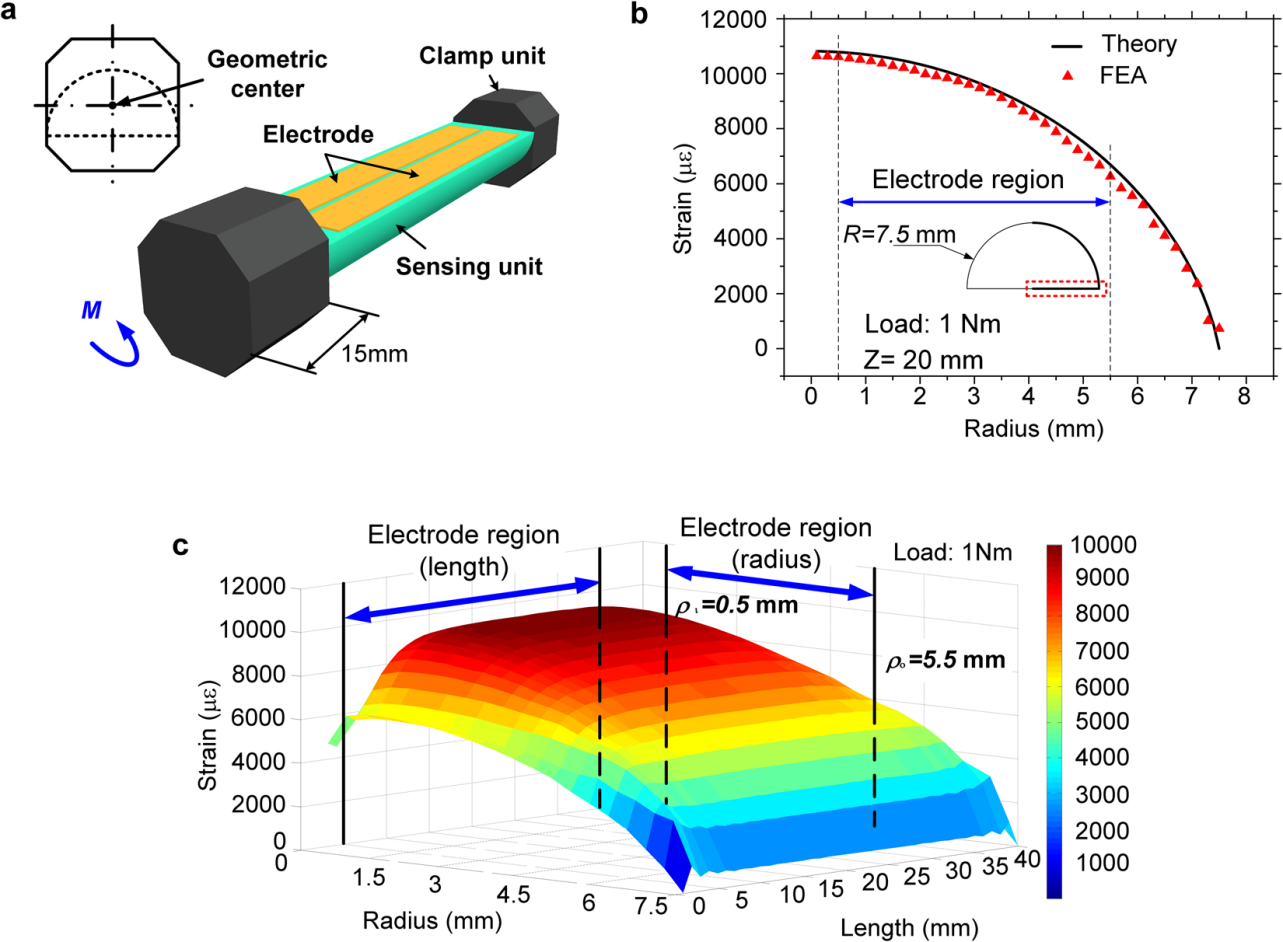
The design of the specimen with its mechanical properties analysis. (**a**) The shape of the specimen with the geometric center and (**b**) the comparison of the theoretical and FFA strain distribution on the section of z = 20 mm, and (**c**) the general strain distribution of the whole measured surface.

The theoretical deduction proves that a shear strain gradient is generated in the half-cylindrical specimen under pure torque. To meet the requirement of Saint Venant’s Principle, a half-cylindrical specimen was machined, and its diameter and length were designed to be 15 mm and 40 mm, respectively (Fig. 2a)^31, 32^.

To cross-check the strain gradient, finite element analysis (FEA) was carried out with ABAQUS. Referring the simulations and the analytical calculations of deformations, Abdollahi *et al*. developed an advanced computational evaluation of the flexoelectric effect in dielectric solids to more accurately evaluate the electro-mechanical coupling effect in micro scales with smooth mesh free methods^35^. According to their research results, the macro-scale and bulk geometric shapes of the specimen induce less coupling. The physical properties of this material, including mass density, Young’s modulus, Poisson’s ratio, and yield stress were measured by ourselves and listed in Table 1, which all agreed with previous reports reasonably^24–26^. The geometric center of the octagonal prisms and the testing specimen coincided, to ensure that a pure torque was imposed upon the testing specimen. Because the experimental frequency range (which is less than 25 Hz) lied far away from the torsional resonance frequencies of the designed specimen (the 3^rd^ mode of frequency of the specimen is calculated as 2664.1 Hz,), a static analysis was considered to be reasonable. Hence, for FEA simulation, a torque of static 1 Nm (59.5% of the upper limit of the elastic range) was imposed upon the bar.

**Table 1:** Measured physical properties of PVDF.

Material	Mass density (kg/m^3^)	Young’s modulus (MPa)	Poisson’s ratio
PVDF	2.1 × 10^3^	1.73 × 10^3^	0.38
Epoxy	2.3 × 10^3^	20	0.38

The shear strain distribution calculated by theory and by FEA on the half cylinder plane of *z* = 20 mm is demonstrated in Fig. 2b. The general strain gradient of the whole plane calculated by FEA is observed inhomogeneous at the edges of the specimen, which is probably due to the edge effects in elastic mechanics (Fig. 2c). In general, the results obtained in both methods are consistent. The most significant discrepancy appears less than 6%, where the FEA output is lower than the theoretical prediction. This tiny difference is proposed to result from the plain hypothesis employed in the theoretical deduction, and the boundary conditions and unit type/size selection used in the FEA simulation.

The electrode areas for polarization measurement were marked in Fig. 2c. The regions on the half cylinder plane where |*ρ*| < 0.5 mm or > 5.5 mm, and |*z* − 20| > 15 were excluded for the sake of non-uniform strain gradient distribution. The *c* in Eqn. 12 is then simplified due to the good linearity, as *c’*:

13 $$c^{\prime} =\frac{{\int }_{{\rho }_{i}}^{{\rho }_{o}}c(\rho ){\rm{d}}\rho }{{\rho }_{o}-{\rho }_{i}},$$

where *ρ*_i_ and *ρ*_o_ are the inner and outer radius of the electrode coated boundary.

The general electric polarization along ϕ direction, which is denoted as *P*ϕ, can be readily computed by measuring the electric charge density on the plane parallel with the *z* coordinate direction and the potential contributions from the residual piezoelectricity:

14 $${Q}_{\varphi }={P}_{\varphi }{A}_{e}+{Q}_{p}={\mu }_{\varphi z\rho \varphi }c^{\prime} M{A}_{e}+{Q}_{p}={\mu }_{2312}c^{\prime} M{A}_{e}+{Q}_{p},$$

where *Q*ϕ, *Q*_p_ and *A*_e_ are the induced general electric charge, the piezoelectricity induced charge, and the coated electrode area, respectively, and then the effective flexoelectric coefficient *μ*_2312_ is calculated from the experimental measurement:

15 $${\mu }_{2312}=\frac{({Q}_{\varphi }-{Q}_{p})}{M}\cdot \frac{1}{c^{\prime} {A}_{e}}.$$

Three identical specimens are measured and the average slope of (*Q*ϕ − *Q*ϕ)*/M* is obtained. The contribution from the residual piezoelectricity is finally ignored due to the lower magnitudes from the general polarizations, based on the discussion below. Hence, the calculated effective flexoelectric coefficient *μ*_2312_ is shown in Table 2. With unsuspected reproducibility, the measured effective tensor is independent of frequency in the investigated frequency range. The precision of the results is ideal. By statistics, the effective flexoelectric coefficient component *μ*_2312_ of PVDF is (1.1 ± 0.1) × 10^−8^ C/m at room temperature, which is in good accordance with the currently reported magnitudes of the other effective flexoelectric coefficient tensor components of the un-polarized PVDF.

**Table 2:** Measured values of flexoelectric coefficient component *μ*_2312_ of PVDF.

Frequency (Hz)	*μ*_2312_ (×10^−8^ C/m)		
	Specimen 1 (C/m)	Specimen 2 (C/m)	Specimen 3 (C/m)
0.5	1.1 ± 0.1	1.1 ± 0.1	1.2 ± 0.1
1	1.1 ± 0.1	1.1 ± 0.1	1.1 ± 0.1
1.5	1.1 ± 0.1	1.1 ± 0.1	1.2 ± 0.1
2	1.1 ± 0.1	1.1 ± 0.1	1.1 ± 0.1

## Discussions

### Potential impacts from residual piezoelectricity

Under the experimental conditions, when pure torque was applied, shear, compressive, and tensile stresses might all exist, and thus polarizations might be generated by not only flexoelectricity, but also piezoelectricity. Therefore it is necessary to evaluate the possible impacts from the residual piezoelectric effects and the induced charge *Q*_*p*_.

**Figure 3:**
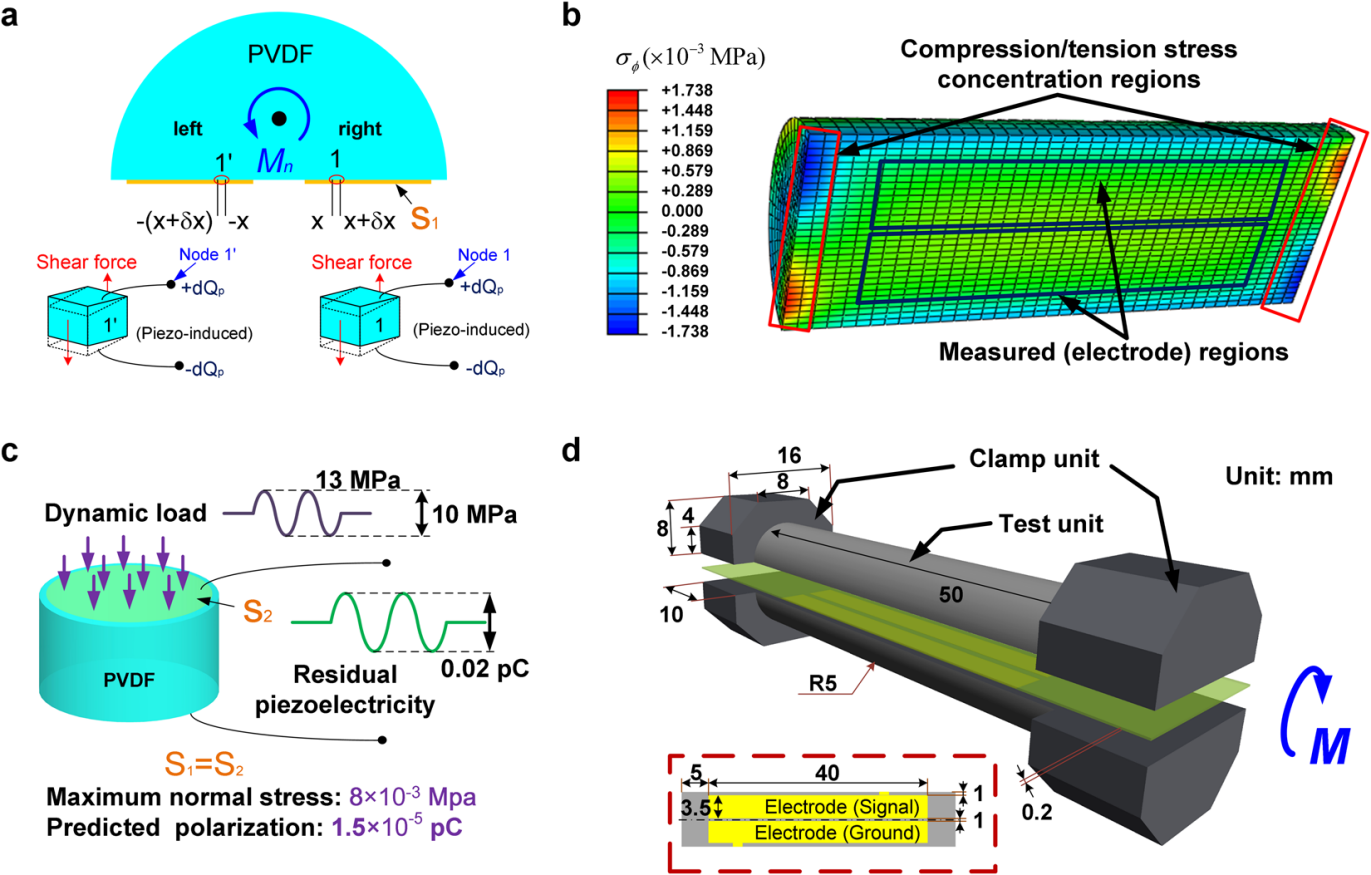
The discussion of the potential residual piezoelectric induced polarization. (**a**) The analysis of the possible impact on the shear stress induced residual piezoelectricity, (**b**) the FEA result of the inhomogeneous compression/tension stress concentration on the specimen, (**c**) the experimental method of the normal stress induced residual piezoelectricity, and (**d**) the sandwich structure of the verification of non-uniformity of strain gradient.

The shear stress induced electric polarization from the piezoelectric effect is analyzed and demonstrated in Fig. 3a. An infinitesimal length from position *x* to *x* + *δx* on the coated electrode is considered. When the shear stress is exerted, electric polarization *δQ*_*p*_ will be induced. Similarly, on the opposite side of the electrode, an equal amount of deformation and electric charge will be generated in the identical length from −*x* to −(*x* + *δx*). Hence, it is apparent that identical amount of electric charges are induced on both electrodes due to the piezoelectric effect. Therefore, although the shear stress-induced polarization due to the piezoelectric effect may alter the bias of the real time waveform, the peak-to-peak value, and thus our measured flexoelectric constant, will not be influenced.

FEA is carried out to assess the magnitude of normal stress (including both compressive and tensile stresses) induced by the applied torque load on the PVDF half-cylindrical bar with the same dimensions of the tested specimens (Fig. 3b). When a torque of 1 Nm is imposed upon the bar, compressive and tensile stresses only exist close to the edges of the specimen (within no more than 1 mm along the *z* direction). In our experiment, the electrodes are not coated in these areas, thus the influence from the compressive and tensile stresses can be largely excluded (Fig. 3c). Moreover, the exact contribution of the compression/tension stress induced piezoelectricity is also measured experimentally. A cylindrical PVDF specimen with uniform cross-section area (Fig. 3b) is fabricated and measured under compressive load. To reduce the measurement error, the compressive load exerted is 10 MPa, which is over 60% of the elastic limit, and its electrode area is identical with that of the half cylinder shape-sectioned specimen. The electric polarization is measured to be approximately 0.05 pC. According to the FEA result, the maximum normal stress of the half cylinder section specimen is 0.008 MPa under the torque. With a linear assumption, 0.008 MPa can only induce a polarization of approximately 1.5 × 10^−5^ pC, 4–5 magnitudes lower than the experimental measurements. Consequently, the polarizations induced by the normal stresses can be ignored.

### The strain gradient non-uniformity

From FEA results (Fig. 3c), the shear strain gradient is discovered not strictly uniform along the *ρ* direction. To verify the potential impact from the non-uniformity of the strain gradient, uniform shear strain gradient is generated by designing another sandwich-structured specimen, which consists of two identical half cylindrical specimens with the electrodes coated in the similar way (Fig. 3d). Epoxy with low stiffness is applied to insulate the electrodes and to glue the two parts together. Because the thickness of the electrode is two orders of magnitude lower than that of the epoxy, the influence of the electrode onto the deformation behavior is ignored. When a small twisting moment is imposed upon the sandwich specimen, shear strain is generated in both PVDF parts and the rectangular epoxy. According to theoretical deduction, the shear angles *ϕ* of the PVDF parts and the epoxy can be calculated using the following equations^30, 31^:

16 $${\varphi }_{c}=\frac{{M}_{c}l}{{G}_{c}{I}_{c}}=\frac{{M}_{c}l}{{G}_{c}\frac{\pi }{32}{D}^{4}},$$

17 $${\varphi }_{r}=\frac{{M}_{r}l}{{G}_{r}{I}_{c}}=\frac{{M}_{r}l}{{G}_{r}\frac{1}{3}h{b}^{3}},$$

where *M*, *I*, *G*, and the subscripts *c* and *r* are the magnitudes of torque, the polar moment of inertia, shear modulus, the cylindrical parts and epoxy, respectively. *l* and *D* represent the length and diameter of the cylindrical bar, while *h* and *b* define the long and short side of the rectangular electrode.

With the applied conditions, we have ${\varphi }_{c}={\varphi }_{r}$ and ${M}_{c}+{M}_{r}={M}_{n}$, where *M*_*n*_ is the torque that is experimentally applied. Substituting these boundary conditions to Eqn. 16 and 17, we have:

18 $${M}_{r}=\frac{32{G}_{r}h{b}^{3}}{3\pi {G}_{c}{D}^{4}}{M}_{c}.$$

The basic properties of PVDF and epoxy are experimentally measured and listed in supplemental materials. The detailed method is described in the Supplementary Information. The shear moduli of PVDF and epoxy are computed to be *G*_*c*_ = 0.63 GPa and *G*_*r*_ = 7.97 MPa using $G=\frac{E}{2(1+\nu )}$, where *E* and *v* are Young’s module and Poisson’s ratio, respectively. With the calculated shear moduli and measured specimen dimensions, it suggests that the torque distributed on the epoxy is less than 1‰ of the one on the PVDF bar. As a result, in the following deductions the contribution from epoxy is ignored.

The shear strain *γ*ϕ_z_ in the effective region of the specimen is expressed as:

19 $${\gamma }_{\varphi z}=\rho \frac{d\varphi }{dz}=\rho \frac{{M}_{n}}{G{I}_{p}},$$

where *I*_p_ is the polar moment of inertia of the PVDF bar. The strain gradient along the *ρ* direction is uniform. Since *M*_*n*_, *G*, and *I*_*p*_ are all constants, Eqn. 1 can be greatly simplified as:

20 $${P}_{\varphi }={\mu }_{2312}\frac{\partial {\gamma }_{\varphi z}}{\partial \rho }={\mu }_{2312}\frac{{M}_{n}}{G{I}_{p}},$$

and thus the effective flexoelectric coefficient *μ*_2312_ can be obtained through:

21 $${\mu }_{2312}=\frac{{P}_{\varphi }}{\frac{\partial {\gamma }_{\varphi z}}{\partial \rho }}=\frac{{Q}_{\varphi }}{{A}_{e}}\frac{{G}_{c}{I}_{p}}{{M}_{n}}.$$

As described in Supplementary Materials, the effective flexoelectric coefficient is measured to be (1.1 ± 0.1) × 10^−8^ C/m in the sandwich specimens, which is identical with the result obtained from the half-cylindrical ones, suggesting that the strain gradient non-uniformity in the half-cylindrical specimens do not impose a severe impact on the flexoelectric coefficient measurement. Moreover, it is more convenient to use the half-cylindrical geometry than the sandwich structure. Firstly, the specimens are easier to fabricate. Secondly, the influence from epoxy can be completely excluded. Finally, the deformation model is simpler.

## Conclusions

In this work, an experimental process to measure the 2312 component of the flexoelectric coefficient is proposed and applied to investigate the flexoelectricity of un-polarized polymer PVDF. It is verified in the cylindrical coordinate system that *μ*_2312_ can be obtained by measuring the electric charge in the axial plane of a half-cylinder shaped bar induced by pure torque. The relationship between the shear strain gradient along the radial direction and the torsional moment is presented through a simple method of elastic mechanics deduction based on the plain stress assumption due to the application conditions. Such hypothesis and theoretical deduction is verified by FEA simulation, in light of the experimentally measured physical parameters. Cyclic torque loading is imposed upon three identical PVDF samples at various frequencies from 0.5 Hz to 2 Hz at room temperature, and in the meanwhile the electric charge is monitored. *μ*_2312_ is thus measured accurately, and it is found a frequency-independent constant in this frequency range. The possible impacts from the piezoelectric effect and strain gradient non-uniformity are proved negligible to the measurement. It is believed that the assessment procedure proposed here, including the deduction, simulation, and measurement, can be applied to a wide range of polymeric materials.

## Methods

Our PVDF material was obtained from GEHR Plastics Inc., and its X-ray diffraction pattern indicates that the un-polarized material was a partly crystalized of mainly *α*- phase, which was generally considered to be a non-polarized phase (Supplementary Materials). The electrode material used in this study was the Humi Seal® 948-06 G, which had excellent electrical conductivity and low attached stiffness.

**Figure 4:**
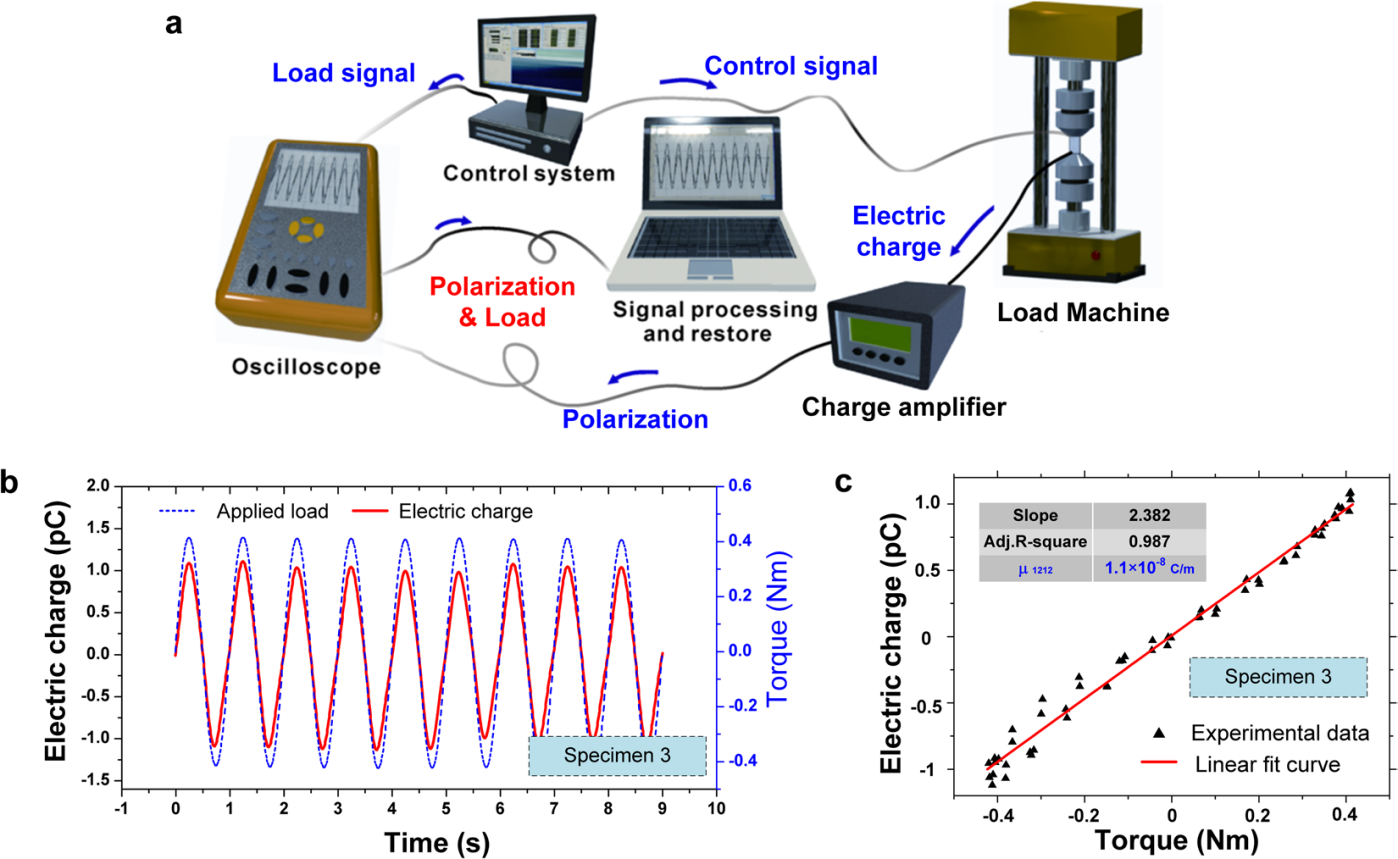
The experiment procedure of the measurement. (**a**) The schematic of the experimental system, (**b**) the real time waveform of the induced polarization with its relating applied torque load measured from the specimen 3, and (**c**) the effective flexoelectric tensor component obtaining procedure.

The experimental setup is demonstrated in Fig. 4a. All tests were carried out at room temperature, which was by far below the reported Curie’s point of PVDF (103 °C)^36^. Dynamic torque load with bias value was generated and imposed with low frequency (MTS ^TM^ 858 testing machine) on the specimen. The cyclic torque *M* in unit of Nm varied as the following sine function with a bias magnitude of 1.0 Nm and amplitude of 0.5 Nm:

22 $$M=1.0+0.5\,\sin (2\pi f\cdot t),$$

where *f* is the load frequency of the dynamic torsional loading. It is worthy to emphasize that the maximum torque (1.5 Nm) is about 30% lower than the elastic limit of the material, and thus it is ensured that all the signals recorded are from elastic response. To exclude the coupled heat from the higher frequency loads on the specimen, the load frequencies are considered to be as low as quasi static. Hence, in this study, the tests were carried out under four various frequencies, where were 0.5 Hz, 1.0 Hz, 1.5 Hz, and 2.0 Hz, respectively.

The electric polarization was measured by amplifying the electric charge induced by the shear strain gradient (B&K-2962A charge amplifier, 1 V/pC setting). The dedicated electric charge shield and grounding design was applied for signal transmission, and the waveforms of the mechanical loading and voltage signals were displayed in real time and recorded using an oscilloscope. The experimentally obtained data is filtered by the MATLAB for further reducing the noises and the potential zero drift (Butterworth 2^nd^ order low pass filter with upper frequency 2 *f* for reducing the noise, and excluding the trend terms from the zero drift). The comparison of the filtering effect is shown as the Supplementary Materials. One of the processed examples is shown in Fig. 4b, with the loading frequency *f* = 1 Hz, on the specimen 3.

For data processing, the strain gradient was calculated from the linear relationship with the torque as expressed in Eqn. 12. The electric polarization was obtained by dividing the charge by electrode area, the (*Q*ϕ − *Q*_p_)*/M* is calculated from Fig. 4c, and then the effective flexoelectric coefficient tensor component*μ*_2312_ is computed and listed in Table 2.

## Electronic supplementary material

Supplementary Information
