# Supplementary material for: Investigation of the 2312 flexoelectric coefficient component of polyvinylidene fluoride: Deduction, simulation, and mensuration
Source: Sci Rep. 2017 Jun 9;7:3134. doi: 10.1038/s41598-017-03403-7 (PMC5466657; doi:10.1038/s41598-017-03403-7)
Supplement: Supplementary file 1 — Supplementary Information [file 41598_2017_3403_MOESM1_ESM.pdf]

Supplemental materials

Shuwen Zhang[1], Kaiyuan Liu[1], Minglong Xu[1,*], Hao Shen[2], Kai Chen[2,*], Bo Feng[1] and Shengping Shen[1]

1. State Key Laboratory for Strength and Vibration of Mechanical Structures, School of Aerospace, Xi'an Jiaotong University, Xi'an 710049, China;
2. Center for Advancing Materials Performance from the Nanoscale (CAMP-Nano), State Key Laboratory for Mechanical Behavior of Materials, Xi'an Jiaotong University, Xi'an 710049 , China

*Author to whom correspondence should be addressed. Email: mlxu@xjtu.edu.cn (M.X.) or kchenlbl@gmail.com (K.C.).

Three issues are presented on this file: one is the applied physics material parameters of PVDF and epoxy, the second is the experimental procedure and effect of the verification of the non-uniformity of the strain gradient, and the last is the effect of the signal processing method.

1. Measurement of the Young's module, the yield stress and the Poisson's ratio of PVDF and epoxy

The quasi-static measurement method is adopted because the viscoelasticity of the materials, and because the applied experiment frequency is in quasi-static condition.

1.1 The Young's module

A MTS®880 load machine is adopted to output the quasi-static linear increased compression load onto the test specimen, and the displacement of the load end is synchronously recorded as the quasi static load varies, seen as Fig. 1. The load-displacement curve is analyzed to calculate the strain and stress of the specimen, and the Young's module is then calculated by

$$E = \frac{\frac{F}{s}}{\frac{\Delta h}{h}}, \tag{1}$$

where *E, F, s, Δh*, and *h* represent the Young's module, the applied force load, the cross-section area, the deformation along load direction, and the total length along the load direction.

In equation (1), *F* is obtained by the load machine, *s* is known from the design of the specimen, *Δh* is obtained from the machine, and *h* is known from the design,

respectively.

To decrease the inhomogeneous stress distribution, the specimen is designed as the cylinder-cross sectioned bars. To ensure the accuracy of the displacement, the stiffness of the load exerting plates are at least 100 times larger than that of the testing materials.

To increase the precision of the measurement, the force load and the displacement is adopted by the difference value. With this method, the Young's module of PVDF is measured as 1.725 GPa, and the Young's module of the epoxy is measured as 21.98 MPa.

1.2 The yield stress of PVDF

To obtain the yield stress, the method presented from section 1.1 is adopted with longer loading time and larger loading amplitudes, to find the slope altering point(s) of the load-displacement curve. Due to the viscoelasticity of PVDF, more than one altering point exists in the experiment. To ensure the elastic range of the flexoelectric measurement, the first altering point is used to ensure the yield stress of PVDF.

With this method, the yield stress of the PVDF is measured as 16.9MPa.

1.3 The Poisson's ratio

When the experiment mentioned in section 1.1, the Keyence® laser sensing system is used to measure the Poisson's ratio, seen as Fig. 1. When the load is exerted, the material deformation is along both the loading direction and the load-vertical direction. The deformation along the load-vertical direction is measured. The Poisson's ration is then calculated as

$$\nu = \left| -\frac{\varepsilon}{\varepsilon'} \right| = \frac{\frac{\Delta h}{h}}{\frac{\Delta d}{d}}, \tag{2}$$

where ε, ε', Δd, and d represents the strain along load direction, the strain along load-vertical direction, the deformation value from the laser sensing system, and the dimension of the load-vertical direction.

With this method, the Poisson's ratio of PVDF and epoxy are both measured as 0.38.

2. The experimental procedure of the verification of the non-uniformity of the strain gradient

The geometric sizes are presented in Figure 2 (a) to show the details of the fabrication and the experimental preparations. The shear strain distribution along the ρ direction of the combined specimen is shown in Figure 2 (b). The fabrications of the two-unattached units of the specimen are shown as Figure 2 (c).

To cross-check the theoretical deduction, FEA was also carried out with ABAQUS to

simulate the torque angle as a function of the torque that was applied on a PVDF-epoxy combined specimen with the same dimensions of the realistic specimen. From Figure 2 (d), it shows that the FEA results coincides with the theoretical deduction well. Furthermore, experimental verifications are also done, and the measured torque angle is just slightly lower than the calculated and simulated values. This tiny difference is proposed to result from the applied boundary conditions and unit type/size selection used in the FEA simulation. Therefore, the torque-angle information is adopted from the theoretical calculation.

To exclude the polarization induced by the inhomogeneous strain gradient at the edge of the testing specimen, the coated electrode areas were also determined and marked in Figure 2 (b) and (c). The regions on the half cylinder plane where $|\rho| < 0.5$ mm or > 4 mm, and $|z - 20| > 5$ were excluded for the sake of non-uniform strain gradient distribution.

Because static electric charge was difficult to measure, low frequency dynamic torque was generated with a MTS-858 testing machine and imposed on the specimen. The cyclic torque M in unit of Nm varies as the following sine function with a peak-to-peak magnitude of approximately 0.82 Nm with no bias value.

$$M = 0.41\sin(2\pi f \cdot t), \quad (3)$$

where f is the frequency of the dynamic torsional loading. It is worthy to emphasize that the maximum torque, ± 0.41 Nm, is about 75% lower than the elastic limit of the specimen, and thus it is ensured that all the signals recorded are from elastic response. In this verification, the tests were carried out under the quasi-static frequency 0.5 Hz, with 5 repeated times on the three of each identical specimen.

The ideal strain gradient was calculated, and then the effective flexoelectric coefficient tensor component μ_{2312} was computed and listed in Table I. By statistics, this effective flexoelectric coefficient component is calculated as $(1.1 \pm 0.1) \times 10^{-8}$ C/m in general, which are identical with the result of the main result in this work.

3. The effect of the signal processing method in this work

The signal processing method in this work is adopted by MATLAB with the Butterworth 2nd order low pass filter with upper frequency $2f$ (load frequency) for reducing the noise, and excluding the trend terms from the zero drift. The filtering effect is seen as Figure 3.

TABLE I. Measured values of flexoelectric coefficient component μ_{2312} of PVDF of the sandwich structure

Repeat times	μ_{2312} ($\times 10^{-8}$ C/m, at 0.5 Hz)		
	**Specimen 1**	**Specimen 2**	**Specimen 3**
1	1.2 ± 0.1	1.2 ± 0.1	1.1 ± 0.1
2	1.2 ± 0.1	1.2 ± 0.1	1.2 ± 0.1
3	1.2 ± 0.1	1.1 ± 0.1	1.1 ± 0.1
4	1.2 ± 0.1	1.1 ± 0.1	1.1 ± 0.1
5	1.2 ± 0.1	1.1 ± 0.1	1.1 ± 0.1

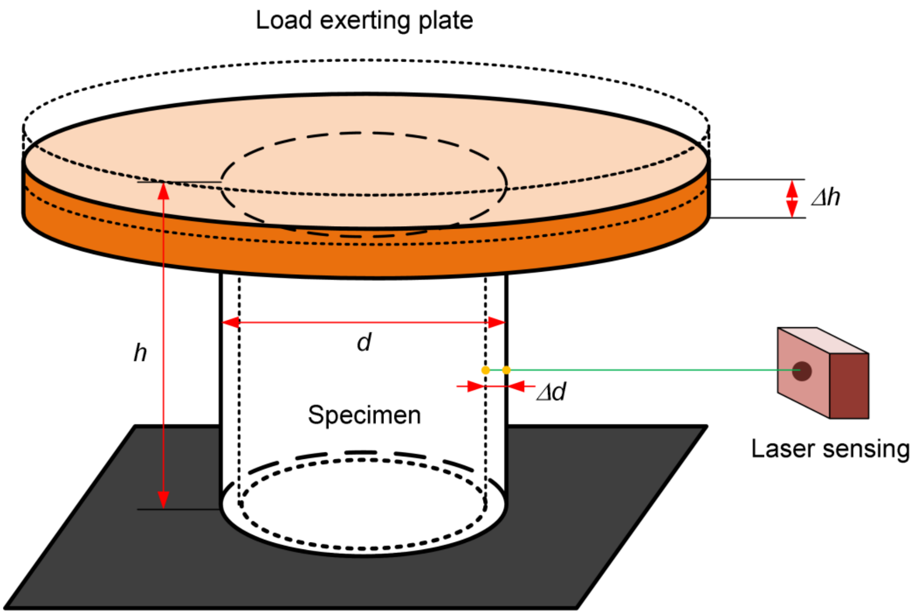

Fig. 1 the schematic diagram of the material measuring system.

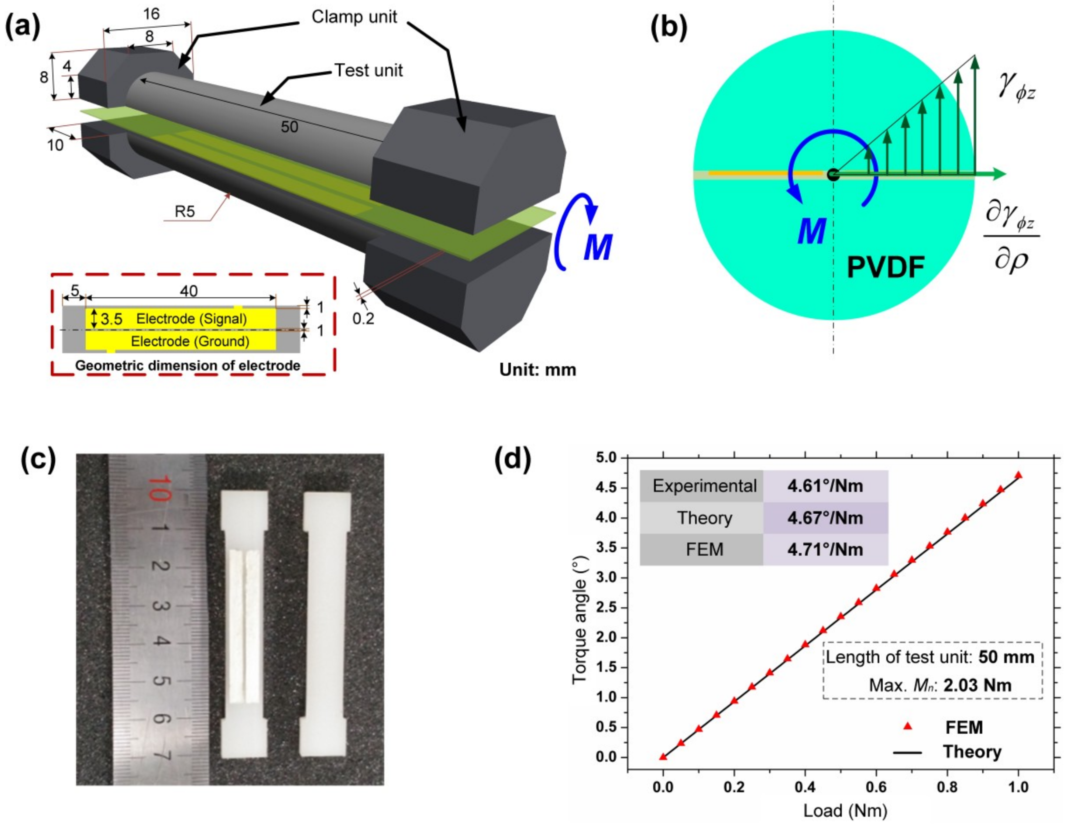

Fig. 2 The schematic of the experiment of the sandwich structure. (a) The ideal shear strain distribution, (b) the shape of the sandwich-shaped specimen with the geometric dimensions, (c) The outlook of the two-unattached units of the specimen, and (d) the mechanical-deformation relationship of the analysis and the experiment.

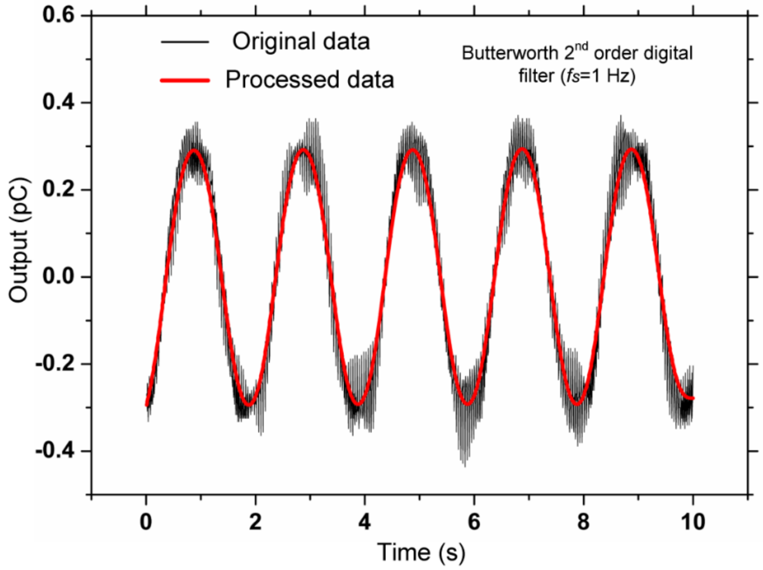

Fig.3 The real time waveform of the original data and the processed data.
